# Editorial: Mathematical and computational methods in physiology

**DOI:** 10.3389/fphys.2022.975075

**Published:** 2022-08-08

**Authors:** Pedro Femia, Juan Melchor, Pedro Carmona-Saez

**Affiliations:** ^1^ Department of Statistics and Operations Research, University of Granada, Granada, Spain; ^2^ Instituto de Investigación Biosanitaria, ibs.GRANADA, Granada, Spain; ^3^ Excellence Research Unit “Modeling Nature”, University of Granada, Granada, Spain; ^4^ Centre for Genomics and Oncological Research (GENYO) Pfizer, Andalusian Regional Government, PTS Granada-Avenida de la Ilustración, University of Granada, Granada, Spain

**Keywords:** mathematical physiology, modelling, omics integration, biostatistical methods, machine learning, computational methods

Physiology constitutes a broad discipline that covers the study of the different hierarchical levels of living organisms, from the cellular one to higher levels, such as tissues or organs. The articles of this monography provide an excellent example of the telescopic capacity of this discipline. A common nexus among all these levels is that physiological systems are epistemologically complex. This implies that their study requires a necessary reduction of the complexity in order to elaborate a formal and manageable description of the system, giving rise to a model. Therefore, we need to develop mathematical models that represent the original system instead of studying it. Nevertheless, even this representation can present an unmanageable degree of complexity. Typically, the description of the physiological processes relies on non-linear relationships among a high number of variables that involve many parameters of unknown value.

However, it is possible to consider different strategies to reduce the descriptive complexity. Functional and temporal simplifications are two of them. Polynomial approximations, for example, can be accurate enough to describe a phenomenon while remaining with sufficient simplicity to treat them mathematically. On the other hand, processes that occur at a different time scale than those under study can be ignored or assumed to be at their stationary values. Aldana et al. have considered these two approaches (Aldana et al.). On the one hand, they propose a discrete model for the human sperm acrosome reaction, and on the other hand, the change of state of the variables involved is synchronous. Changes in physiological processes are continuous and smooth events, but typical non-linearities such as saturation and synergism can generate switching behaviours if the stimulus varies appropriately. Therefore, assuming categorical values for the state variables can be an efficient reductionist approach in a proper context, and as a result, the representation of functional relationships is more manageable. Synchronization implies that the model ignores the transition between states, focusing on the state space’s particular configuration reached at the end. But the capacity of this model to reproduce many of the results found in the literature supports these restrictions.

At this point, it is good to remind the famous aphorism attributed to the prestigious British statistician George E.P. Box, which says that all models are wrong, but some are useful. Effectively, the reductionism intrinsic in any model supposes that it is impossible to simultaneously optimize generality, realism and precision. The suitability of any model depends on the aspect of reality it retains.

But once a model is built, the challenge is to progressively remove restrictions and simplifications to bring this representation closer to the phenomenon under study. The article presented by Afshar et al. illustrates this aspect (Afshar et al.). They have considered new processes to extend a previous model about glucose uptake in the enterocyte. As a result, they provide a more accurate description of the role of apical and basal GLUT2 activity on intestinal glucose transport. In this vein, Napoli et al. Warn that the traditional sinusoidal approach to the modelization of the breathing cycle is too imprecise, and it leads to hypotheses and results that are not correct. For example, sinusoidal models of breathing have been used to calculate the expected inhaled mass breathing on nebulizers or to estimate the social distancing in indoor environments. After Napoli et al.’s paper, these inferences probably need some reviewing (Napoli et al.).

The battle to overcome descriptive complexity finds its best ally in the optimization of computational methods. Their permanent improvement makes it possible to apply powerful numerical techniques that reproduce the system’s behaviour even when it is impossible to do a complete analytical treatment. For example, the characterization of the scapholunate’s functioning in Marqués et al. is based on the representation of the complex geometry of the joint using the finite element method from a segmetation image proccessing (Marqués et al.). It is a procedure that allows obtaining an accurate approximation to the solution of the biomechanical behaviour in terms partial differential equations that governs the physiological system. Typically, these equations involve such a degree of complexity that analytical methods cannot solve them. The finite element method discretizes the original description into a representation that admits a numerical solution.

Branen et al.’s paper present a multi-objective optimization procedure based on machine learning methods (Branen et al.). They aim to show that it is possible to design a model-based optimal predictive control strategy to optimize the vagus nerve stimulation to achieve the desired heart rate and blood pressure response. As simulation studies allow for modifying the system conditions in a way impossible in the natural system, the in-silico experimentation can represent the first step toward experimental or clinical implementation of the tested protocol. Today, modelling in physiology already has a great tradition. The first models developed under this discipline have constituted a source of inspiration to build improvements or new modelling strategies that bring us closer and closer to the system they try to represent. Mathematical and computational methods are not only powerful tools to deal with analytical barriers, but they are also an innovation engine that opens new and unsuspected possibilities. However, overcoming the obstacles imposed by life’s complexity is still a challenging field.

